# Basal Cell Carcinoma with Osteoma Cutis

**DOI:** 10.7759/cureus.3170

**Published:** 2018-08-21

**Authors:** Stella X Chen, Philip R Cohen

**Affiliations:** 1 School of Medicine, University of California, San Diego; 2 Dermatologist, San Diego Family Dermatology, San Diego, USA

**Keywords:** basal, bone, calcium, calcinosis, carcinoma, cell, cutis, ossification, osteoma

## Abstract

Osteoma cutis is the formation of bone within the skin. It can present as either primary osteoma cutis or secondary osteoma cutis. Secondary osteoma cutis is more common and is associated with inflammatory, infectious, and neoplastic disorders, including basal cell carcinoma. A 79-year-old Caucasian man without underlying kidney disease or calcium abnormalities presented with a basal cell carcinoma with osteoma cutis on the chin. Basal cell carcinoma with osteoma cutis has seldom been described; however, the occurrence of this phenomenon may be more common than suggested by the currently published literature. The preferred treatment is surgical excision—with or without using Mohs micrographic technique. When the histopathologic examination reveals bone formation in the skin, clinicians should consider the possible presence of an adjacent malignancy, such as a basal cell carcinoma.

## Introduction

Basal cell carcinoma is the most common type of skin cancer. Osteoma cutis is the formation of bone within the skin; it can occur either as a distinct cutaneous entity or associated with other conditions. In primary osteoma cutis, idiopathic ossification occurs and can be congenital, acquired, or related to Albright’s hereditary osteodystrophy and fibrodysplasia ossificans progressiva. Secondary osteoma cutis is more commonly seen and may be associated with infectious, inflammatory, or neoplastic disorders, including basal cell carcinoma. Individuals who have a basal cell carcinoma with osteoma cutis have not been frequently reported. The tumors primarily involve the face in elderly, light-skinned individuals. A 79-year-old man with basal cell carcinoma-associated osteoma cutis is described and other conditions associated with cutaneous ossification are summarized.

## Case presentation

A 79-year-old man with a history of prior basal cell carcinoma presented with a new lesion on the right chin. His other medical conditions included acid reflux, osteoarthritis, seasonal allergies, and spinal stenosis; his medications included fexofenadine 60 milligrams twice daily and lansoprazole 15 milligrams daily.

**Figure 1:**
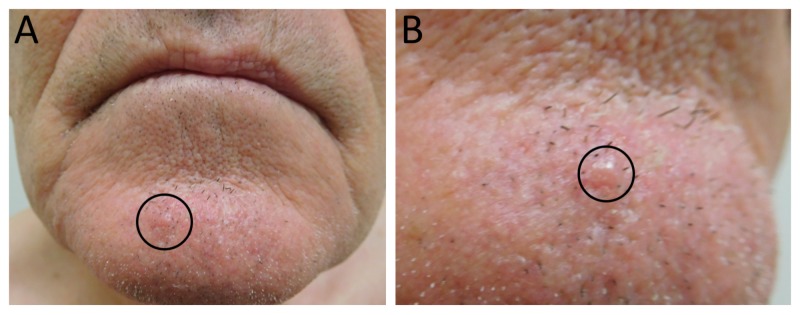
Basal cell carcinoma with osteoma cutis Distant (A) and closer (B) views of a basal cell carcinoma with osteoma cutis (black circle) presenting as a 3-millimeter skin-colored, pearly papule on the chin of a 79-year-old Caucasian man.

**Figure 2:**
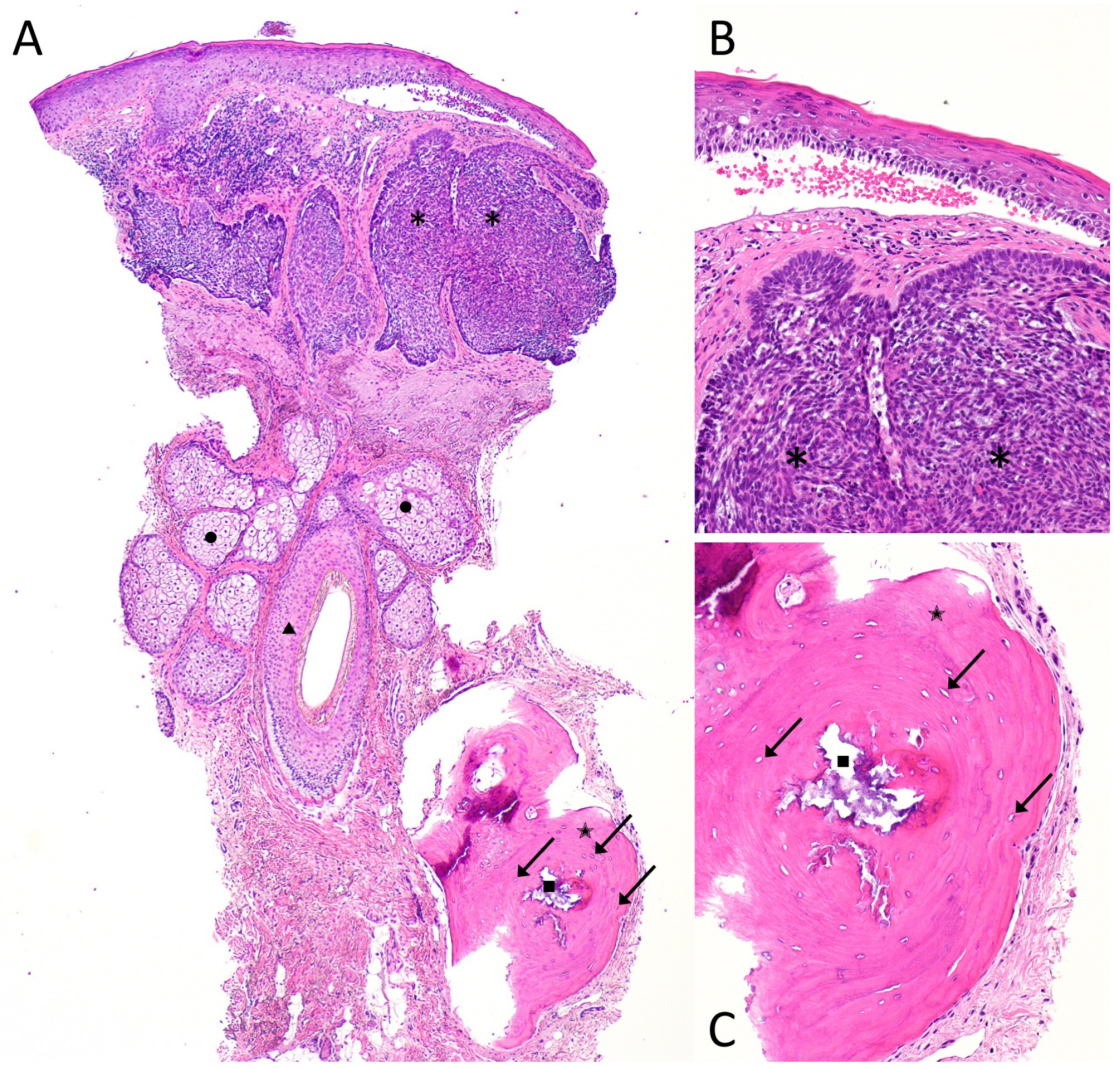
Pathologic features of basal cell carcinoma with osteoma cutis from a punch biopsy of the chin Distant (A) and closer (B and C) views of microscopic findings observed on the hematoxylin and eosin stained sections from a punch biopsy of basal cell carcinoma with osteoma cutis from the chin of a 79-year-old Caucasian man. There are nodular aggregates of basaloid tumor cells (A and B) in the upper reticular dermis (*). In the deeper dermis (A), there is a prominent hair follicle (triangle) and its associated sebaceous glands (circles). Also present in the deeper dermis (A and C) is bone (star). The bone (A and C), representing osteoma cutis, has a central Haversian canal (square) and numerous osteocytes (arrows). (Hematoxylin and eosin: A, x4; B, x20; C, x20).

The physical examination revealed a tender, pearly, skin-colored 3-millimeter papule on the right chin (Figure 1). A provisional diagnosis of basal cell carcinoma was rendered and a punch biopsy was performed. A histologic examination of hematoxylin and eosin stained sections revealed an atypical basaloid keratinocyte proliferation in a nodular configuration with associated inflamed fibromyxoid stroma, and a subjacent aggregate of osteoid in concentric whorls (Figure 2). The patient had normal calcium levels and kidney function and did not have a history of acne with scarring.

The correlation of clinical presentation and histopathologic findings established a diagnosis of nodular basal cell carcinoma with osteoma cutis. The tumor and associated bone were excised using Mohs micrographic surgery, which is a surgical technique that incorporates the microscopic examination of excised margins to ensure the complete removal of cancerous cells while sparing as much normal skin as possible. It is the most effective technique for the removal of squamous and basal cell cancers. In our patient, there has been no recurrence to date.

## Discussion

There are several morphological subtypes of basal cell carcinoma that include nodular, superficial, morpheaform or infiltrative, and pigmented. The typical clinical presentation involves a skin-colored papule with overlying telangiectasias that may bleed easily. Larger lesions may ulcerate and developed rolled borders [1].

The diagnosis of basal cell carcinoma is established by the microscopic evaluation of a biopsy. Histologic findings include basaloid keratinocytes with peripheral palisading nuclei and surrounding stromal retraction. Histologic findings reflect the aggressive and non-aggressive behaviors of clinical subtypes. Non-aggressive patterns include superficial and nodular subtypes, whereas aggressive patterns include the morpheaform or infiltrative and micronodular subtypes [2].

Occasionally, the basaloid tumor cells or surrounding stroma contain associated pathologic elements such as amyloid or bone. The formation of bone in dermal and subcutaneous tissue is known as osteoma cutis. Other names for this condition include cutaneous ossification, military osteoma, and osteosis cutis [3].

Bone mineralization within the skin can occur as a primary or secondary phenomenon. Primary osteoma cutis occurs idiopathically and accounts for 15 percent of cutaneous mineralization processes. Secondary osteoma cutis is associated with other medical conditions and comprises the remaining 85 percent of cutaneous ossification [3].

Primary osteoma cutis occurs idiopathically in tissue without underlying abnormalities or calcification and presents with four syndromes: Albright’s hereditary osteodystrophy, fibrodysplasia ossificans progressiva, plate-like osteoma cutis (congenital or acquired), and progressive osseous heteroplasia (Table 1) [4-12]. Secondary osteoma cutis is associated with several medical conditions, including acne, folliculitis, lupus erythematosus, and other disorders summarized in Table 1. Rarely, bone formation can be seen in cutaneous malignancy, including in basal cell carcinoma.

**Table 1:** Associated conditions in patients with primary and secondary osteoma cutis

Osteoma Cutis	Reference
Syndromes associated with primary osteoma cutis	[4-5]
Albright’s hereditary osteodystrophy	[4-5]
Fibrodysplasia ossificans progressiva	[4-5]
Plate-like osteoma cutis	[4-5]
Progressive osseous heteroplasia	[4-5]
Medical conditions associated with secondary osteoma cutis	[8]
Infectious or inflammatory conditions	[8]
Acne vulgaris	[8]
Chronic venous insufficiency	[8]
Dermatomyositis	[8]
Folliculitis	[8]
Lupus erythematosus	[8]
Morphea	[8]
Scleroderma	[8]
Syphilis	[8]
Benign neoplasms	[7-8]
Chondroma	[8]
Chondroid syringoma	[8]
Desmoid tumor	[8]
Dermatofibroma	[8]
Epidermal nevus	[8]
Hemangioma	[8]
Infundibular cyst	[8]
Lipoma	[8]
Melanocytic nevus	[7-8]
Neurilemmoma	[8]
Pilar cyst	[8]
Pilomatricoma	[7,8]
Pyogenic granuloma	[8]
Trichoepithelioma	[8]
Trichofolliculoma	[7-8]
Malignancy	[6-12]
Atypical fibroxanthoma	[7-8]
Basal cell carcinoma	[6-12]
Bronchogenic carcinoma (metastatic)	[8]
Infantile fibromatosis	[7]
Malignant melanoma	[7-8]
Mixed tumor of the skin	[7]
Other	[7-8]
Actinic keratosis	[8]
Gardner’s syndrome	[8]
Myositis ossificans	[8]
Scar	[7]
Trauma	[8]

In our patient, bone formation was observed adjacent to a basal cell carcinoma on the chin. He did not have pre-existing syndromes listed in Table 1, such as Albright’s hereditary osteodystrophy or fibrodysplasia ossificans progressiva, nor did he have trauma to the area or dermatological conditions such as acne or folliculitis. Due to the immediate proximity to a basal cell carcinoma, the man was diagnosed with secondary osteoma cutis that was associated with basal cell carcinoma.

Basal cell carcinoma with osteoma was initially described by Roth et al. over 50 years ago; they reported 10 individuals whose basal cell carcinoma had cutaneous ossification [8]. Since the original description, only a few case series and individual reports have been published describing bone formation as an incidental finding in basal cell carcinoma [9-11].

Previously reported patients with basal cell carcinoma and associated osteoma cutis had tumors that were most commonly located on the face and contained bone fragments ranging 0.1 to 18 millimeters in size. Shoji et al. reported a 66-year-old man with bone formation that was more than 5 millimeters in size involving a basal cell carcinoma on the occipital scalp [7]. Most recently, Boyd et al. described five patients with osteoma cutis in nodular or micronodular basal cell carcinoma: two of the cancers were on the face and three were on the trunk, unlike our patient whose tumor occurred in the right chin [6].

The incidence of basal cell carcinoma with osteoma cutis is unknown. However, Boyd et al. observed five individuals during a six-month period [6]. Therefore, this phenomenon may be more prevalent than reported.

Basal cell carcinoma with osteoma cutis—based on the isolated case reports and case series in the literature—does not have a sexual predilection and primarily occurs in elderly, light-skinned individuals as in typical basal cell carcinoma. It most commonly involves the face, similar to our patient who had a basal cell carcinoma located on his right chin. There was one individual, a 77-year-old woman, whose basal cell carcinoma with osteoma cutis formation developed within a pre-existing dermatofibroma on the leg [12].

The presence of osteoma cutis in basal cell carcinomas is not visually detectable since the bone formation is typically located in the deep dermis or subcutaneous fat, similar to our patient. A microscopic examination of these tumors reveals osteoblasts, osteocytes, and fragments of well-developed lamellar bone—with or without calcifications—typically at either periphery of the tumor or within the surrounding stroma; less commonly, these features may be located in the tumor itself or within the adjacent pilosebaceous units. Other pathologic features that may be present include bone marrow elements, melanin deposition, and hemosiderin [6-7].

The pathogenesis of both primary and secondary osteoma cutis remains to be established. Primary osteoma cutis is thought to involve mesenchymal cells, specifically fibroblasts. A preferred theory involves the osteoblastic metaplasia of mesenchymal cells. However, a second theory considers the aberrant migration of normally differentiating mesenchymal cells [13].

Secondary osteoma cutis is associated with several medical conditions, including connective tissue disease, inflammatory conditions, trauma, and tumors (Table 1) [6-12]. Consequently, the pathogenesis is likely to depend on the associated condition. Inflammation or fibrosis of pilosebaceous units may be involved since patients may have ossification of hair follicles; in addition, osteoma cutis can also present in acne or folliculitis [6].

Ossification occurred in prior sites of electrodessication and curettage in three of the patients described by Boyd et al. [6]. Myofibroblasts, which are mesenchymal cells involved in fibrosis and wound healing, produce not only transforming growth factor-beta (TGF-β) but also bone morphogenetic proteins (BMPs); these products can trigger the osteogenesis of primary mesenchymal cells [6]. TGF-β expression is increased in wounds and malignancies, including basal cell carcinomas, so increased production of this growth factor may lead to overlying or adjacent ossification [14]. Boyd et al. also reported an individual in which a patient developed osteoma cutis in a basal cell carcinoma at a different site that had not been previously treated; their observation suggests that ossification may involve a systemic component [6].

The treatment of basal cell carcinoma with osteoma cutis involves the removal of the primary neoplasm. Excision should be performed down to the level of the fat with the removal of osteoma cutis in the surgical specimen, especially for tumors located on the face. Indeed, surgical removal via standard excision or Mohs micrographic surgery (as in our patient) may be preferred over local destruction to prevent wounding or scarring that may lead to further calcification or ossification, especially since there is an increased prevalence of osteoma cutis in sites of prior electrodessication and curettage.

The discovery of bone formation in the skin should alert the clinician to consider the possibility of an adjacent neoplastic or inflammatory entity. However, the prognosis of basal cell carcinoma is not altered by the concurrent presence of osteoma cutis. Therefore, treatment in patients who have the disease should be appropriate for the associated skin cancer.

## Conclusions

Osteoma cutis is the development of bone within the skin and can occur as a primary phenomenon or associated with other disorders. Our patient was a 79-year-old Caucasian man without an underlying kidney disorder or acne scarring, who was found to have adjacent bone formation in a nodular basal cell carcinoma. Although the incidence of osteoma cutis in basal cell carcinoma is unknown, it is likely to be more prevalent than the number of published studies would suggest. The pathogenesis of osteoma cutis associated with basal cell carcinoma remains to be definitively established; however, the production of TGF-β due to inflammation, scarring, or neoplastic cells may be involved. The preferred management of basal cell carcinoma with osteoma cutis is surgical excision with or without microscopically controlled margins; electrodessication and curettage should be avoided since it may predispose to subsequent bone formation in the dermis or subcutaneous fat. If bone formation is observed on histological analysis, clinicians should consider a possible malignant or inflammatory disorder in the surrounding skin.
